# THE SOCIAL STRATIFICATION OF INFORMAL CAREGIVING ARRANGEMENTS IN EUROPE

**DOI:** 10.1093/geroni/igad104.3665

**Published:** 2023-12-21

**Authors:** Marco Albertini, Francesca Zanasi, Giorgio Piccitto

**Affiliations:** Alma Mater Studiorum - Università di Bologna, Bologna, Emilia-Romagna, Italy; Alma Mater Studiorum - Università di Bologna, Bologna, Emilia-Romagna, Italy; Alma Mater Studiorum - Università di Bologna, Bologna, Emilia-Romagna, Italy

## Abstract

In ageing societies, the increasing quota of older and frail individuals creates unprecedented needs for care. As care is often costly and not adequately covered by the welfare state, care responsibilities for older individuals fall on the shoulders of family members. The study of informal care provision is acquiring centrality both in the social sciences and policy discourse, since the care load can bear negative consequences on a range of outcomes, from health (e.g., the “caregiver burden”) to employment. It is important to gain a better understanding of which individuals are the most likely to provide informal caregiving and face its consequences. In the present study, we explore the educational and income differences in the probability of providing informal caregiving to individuals living outside the household in Europe, using the Survey of Health, Aging and Retirement in Europe (SHARE, 2004-2020). As a contribution to the field, we aim at uncovering the mechanisms behind the socio-economic gradient in caregiving, such as differences in health and longevity and time constraints (due to employment and other care responsibilities) that are unequally distributed across social layers. Results show that individuals with tertiary education, and at the top of the income distribution, are more likely to provide care, net of several other factors. The study concludes with attempted explanations of the results, related to the fact that lower educated, and lower income individuals, can more often rely on publicly provided services and means-tested benefits.

